# The Ketogenic Diet Revisited: Beyond Ketones

**DOI:** 10.3389/fneur.2021.720073

**Published:** 2021-07-30

**Authors:** Jo Sourbron, Karin Thevissen, Lieven Lagae

**Affiliations:** ^1^Department of Development and Regeneration, Section Pediatric Neurology, University Hospital Katholieke Universiteit Leuven, Leuven, Belgium; ^2^Centre of Microbial and Plant Genetics, Department of Microbial and Molecular Systems, Katholieke Universiteit Leuven, Leuven, Belgium

**Keywords:** epilepsy, ketogenic diet, mechanism, endocrine system, epigenetics, gut microbiome, serine synthesis, phosphoglycerate dehydrogenase

## Introduction

Epilepsy is a neurological disease characterized by seizures, which affects up to 65 million people worldwide (1). About two-thirds of patients with epilepsy are able to achieve seizure control with current antiseizure medication (ASM) (2), whereas one-third of epilepsy patients are difficult to treat, i.e., patients with drug-resistant epilepsy (DRE). In addition, ASM can induce (serious) adverse events and a significant reduction of the quality of life (QoL), leading to ASM retention rates around 50% (3).

DRE can induce neurobiochemical alterations and emotional and physical dysfunctions. The multifaceted status of DRE patients underscores the emphasis on non-pharmacological options, and therapies that target multiple mechanisms are likely to be more effective to treat DRE (4), thereby acting as a “magic shotgun” rather than a “magic bullet.” If epilepsy surgery is not an option in a patient with DRE, vagus nerve stimulation (VNS) (5) or dietary treatments, such as the ketogenic diet (KD), are valuable alternative options (5–7). Initial studies with dietary treatments report on the classical KD, consisting of 80% fat and 20% protein plus carbohydrate (4:1 KD) or 75% fat and 25% protein plus carbohydrate (3:1 KD) (8). A KD using medium-chain triglycerides (MCTs) leads to more ketones/kcal of energy and a more efficient absorption (9). Therefore, the MCT diet is less restrictive since it consists of a lower amount of fat and a higher intake of protein and carbohydrate (10). The modified Atkins diet (MAD) (11) and the low-glycemic index treatment (LGIT) (12) are other dietary therapies mimicking the seizure reduction result of the KD, but they are less restrictive.

Clinical studies show that both modalities (VNS and KD) lead to a seizure frequency reduction (SFR) by at least 50% in half of the DRE patients. A recent study proposed a treatment algorithm for pediatric DRE, including non-pharmacological treatment options such as VNS and the KD (13).

Interestingly, the KD therapy has some advantages in comparison to VNS: the SFR is slightly higher for patients on the KD (14); the KD is non-invasive, and there are few to no neurotoxic effects when compared to multiple ASM (6). Nevertheless, there are barriers and disadvantages in putting the KD into practice, such as palatability issues, compliance issues, side effects (usually mild), variable response rates, and restrictions to the daily life of the patient (15). Overall, a multidisciplinary team (pediatric neurologist, dietician/nutritionist, and a primary care-giver) is indispensable when dietary treatments are initiated and also during maintenance (16).

Currently, we are unable to pinpoint the mechanism(s) of action of the KD, and it is possible that dietary therapies will be classified as “magic shotguns” (17–20). Therefore, our aim was to elaborate on the newest pathways involved, such as the gut microbiome and serine synthesis.

## Primary Antiseizure Mechanisms of the Kd

A high-fat low-carbohydrate KD replicates a “fasting state.” Subsequently, this state results in (1) fatty acid oxidation producing ketone bodies (KBs), (2) production of polyunsaturated fatty acids (PUFAs), (3) decreased activity of lactate dehydrogenase (LDH), and (4) inhibition of the mTOR pathway (17, 21).

First, KBs [i.e., β-hydroxybutyrate (BHB), acetone, and acetoacetate] have been considered the key effectors of the antiseizure effects of the KD by modulating neurotransmissions and altering metabolic, inflammatory, and epigenetic pathways, as reviewed elsewhere (17, 22–26). BHB, one of the KBs, can also enhance oxidative brain metabolism, resulting in the production of gamma-aminobutyric acid (GABA) (27), GABA_B_ activation (24), the induction of synaptic recycling of glutamate loaded vesicles (24, 28), activation of K_ATP_ (18), activation of two-pore domain potassium channels (K2P) (20), and the decrease of acid-sensing channels (ASICs) (29), thereby dampening neuronal excitability (20). The effects of the KD on neurotransmission have been validated by clinical studies in which patients on the KD therapy were found to have increased GABA levels in the cerebrospinal fluid (CSF) (20). Second, the KD-induced increase of PUFAs can decrease neuronal excitability as well (16, 17, 23, 25, 30). Third, the decreased glucose availability of the KD leads to a significant reduction of LDH activity, resulting in neuronal hyperpolarization and decreased seizures. These features were proven to be part of the antiseizure mechanism of action of stiripentol (24, 31). Fourth, the KD can inhibit the mTOR pathway, which (1) can affect epileptogenesis (26, 32) and (2) can reduce the hyperactivation that has been implicated in patients with tuberous sclerosis complex (TSC), cortical developmental malformations, and DRE (33).

Besides replicating a “fasting state,” resulting in production of various effectors, the antiseizure mechanisms of the KD are also thought to result in anti-inflammatory and antioxidant activity, as reviewed by Koh et al. (15). The nutritionally regulated transcription factor peroxisome proliferator activated receptor gamma, *PPAR*γ, regulates genes involved in anti-inflammatory and antioxidant pathways. The findings of Simeone et al. indeed implicate brain *PPAR*γ*2* among the mechanisms by which the KD reduces seizures (34). More specifically, Knowles et al. found that *in vivo* treatment of rats with a KD increased hippocampal catalase mRNA and protein and that this upregulation required *PPAR*γ*2* (35). Hence, it seems that the KD regulates catalase expression through *PPAR*γ*2* activation and that catalase may contribute to the antiseizure efficacy of the KD.

## Ancillary Antiseizure Mechanisms of the Kd

**Figure 1:**
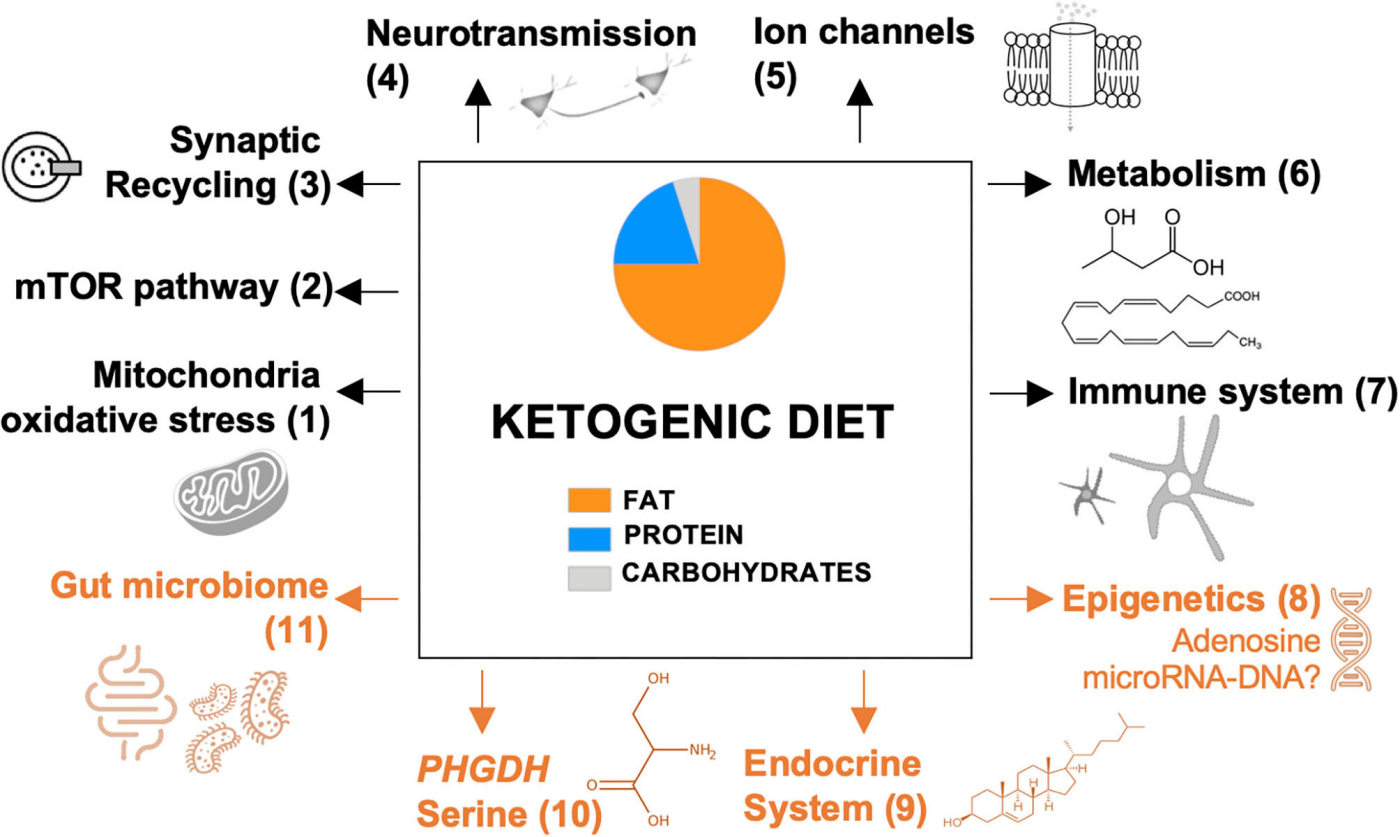
Proposed mechanisms of the ketogenic diet. Previously reported pathways are presented in black; recently discovered and novel pathways are presented in orange. Each pathway is numbered, which refers to the reviewing literature focusing on this specific pathway: (1) Gavrilovici and Rho (23); (2) Danial et al. (26); (3) D'Andrea Meira et al. (24); (4) Gavrilovici and Rho (23); (5) Rho (25); (6) Boison (17) and Gavrilovici and Rho (23); (7) Koh et al. (15); (8-11) this review. mTOR, mammalian target of rapamycin; *PHGDH*, phosphoglycerate dehydrogenase.

In Figure 1, we provide an overview of ancillary mechanisms of action of the KD. These include KD-induced alterations of the endocrine system, gut microbiome, epigenetic mechanisms, and expression of phosphoglycerate dehydrogenase (PHGDH), the first and rate-limiting enzyme of the *de novo* serine biosynthesis pathway. Hence, it seems that multiple mechanisms can be induced by the KD and that these might not be mutually exclusive.

### Production of Neurohormones

The KD therapy can significantly increase the production of certain neurohormones, such as leptin and cortisol (36, 37). First, leptin receptors are found throughout the brain, and their stimulation leads to antiseizure effects by decreasing pro-inflammatory cytokines (e.g., IL-1β), increasing an endogenous anticonvulsant (galanin), and acting as an antioxidant by increasing glutathione and decreasing malondialdehyde (37–39). Second, ghrelin, neuropeptide Y, galanin, and cortisol can induce alterations in GABA uptake and serotonin turnover and affect ion channels, thereby decreasing neuronal excitability, although the exact mechanisms need to be explored by future research (20, 40). Third, cortisol is part of the hypothalamic–pituitary–adrenal (HPA) or stress axis, and targeting this axis can decrease seizures and stress-related comorbidities, e.g., anxiety and depression (41). Consequently, the KD can have beneficial effects in patients with other neurological diseases, diabetes, obesity, reproductive disorders, and other endocrine diseases (42, 43).

### Epigenetic Mechanisms

The KD was found to target epigenetic mechanisms in several rat models of epilepsy, potentially by increasing adenosine (17, 44) that alters DNA methylation and thereby the expression of genes involved in epileptogenesis, such as the purine ribonucleoside adenosine that functions as a homeostatic regulator of DNA methylation (45). This latter study also correlated the epigenetic mechanisms to the antiseizure activities. In addition, pre-clinical data show that the glucose analog 2-deoxy-D-glucose (2-DG) that inhibits glycolysis and thereby mimics the KD decreases the expression of brain-derived neurotrophic factor (BDNF) and the principal receptor, TrkB, an important repressor of neuronal genes *via* NRSF (neuron restrictive silencing factor) induction (46). Thus, a biochemical glycolysis interruption leads to downstream modulation of gene transcription and epileptogenesis (47). Finally, it has been suggested that the KD can change the expression of microRNAs (e.g., mRNA expression decrease of inflammatory interleukines such as IL-1β and IL-6) (15) and of multiple brain genes such as an upregulation of GABA type A receptor subunit alpha 1 (*gabra1*) (20). However, the evidence is currently not strong enough to unequivocally link the expression of these genes with the observed antiseizure effects.

### Gut Microbiome

The study of Newell et al. is the first study to show that the KD affects the gut microbiome (48). The KD therapy inevitably reduces carbohydrate intake and thereby reduces *Faecalibacterium, Blautia* bacteria, *Bifidobacterium*, and *Eubacterium rectale*. The first two bacteria induce fermentation (49) and an elevation of GABA in the hippocampus (50). The latter two bacteria also affect acetate and lactate levels and are involved in regulating the pH and pathogen growth (51). Hence, it seems that specific bacteria can modulate the production of inhibitory neurotransmitters like GABA, possibly by increasing ketogenic gamma-glutamylated amino acids that are substrates for GABA synthesis, which was found to be correlated to the antiseizure effects (52).

Olson et al. were the first to demonstrate that the antiseizure effects of the KD were induced by enrichment of *Akkermansia muciniphila* and *Parabacteroides* populations in the gut microbiome in two distinct murine models of different epilepsy types. Furthermore, transplantation of the KD gut microbiota and suppletion of *Akkermansia muciniphila* and *Parabacteroides* decreased seizure frequencies even in mice on a normal diet (50).

Clinical data also show that the KD influences gut microbiota composition in children with DRE, resulting in increased levels of *Bacteroidetes* (53, 54) and proteobacteria (51). However, these studies were not able to elucidate how microbiome alterations correlate to the antiseizure effect of the KD.

To unravel the correlation between the human gut microbiome and neurological diseases including epilepsy, metagenomics holds great potential (55). Understanding how diet can manipulate seizures may suggest novel therapies. In this respect, probiotics could constitute an alternative therapy as suggested by small clinical studies (56).

### Phosphoglycerate Dehydrogenase and the Serine Synthesis

The KD has recently been shown to induce the expression of genes involved in the serine synthesis, such as PHGDH, in the liver and cerebral cortex of mice (57). The low content of proteins in the KD can result in amino acid stress and thereby induce serine (amino acid) synthesis as a feedback mechanism. In addition, the low amount of glucose of the KD reduces the content of glycolytic intermediate 3-phosphoglycerate (3-PG), which is a substrate of serine synthesis. Henceforth, these two compensating mechanisms can induce the expression of serine synthesis genes, including PHGDH (57) (Supplementary Figure 1).

To date, there are several findings underlining the antiseizure effects of PHGDH activation. First, PHGDH activity is linked with normal brain function. L-serine (synthesized *via* PHGDH) is a key rate-limiting factor for maintaining steady-state levels of D-serine in the adult brain (58). Hence, L-serine availability in mature neuronal circuits determines the rate of D-serine synthesis in the forebrain and controls *N*-methyl-D-aspartate (NMDA) receptor function at least in the hippocampus (59). Second, PHGDH malfunctioning/deficiency is associated with DRE (60), and mice with reduced PHGDH expression, induced by a high-lard-content diet resulting in fatty liver disease, have a severe pre-disposition for development of seizures, more specifically increased seizure episodes and decreased seizure thresholds (61). Third, PHGDH activity is linked to anti-inflammatory action. PHGDH has been identified as a key enzyme for steering macrophage polarization toward an anti-inflammatory M2 state (62). Hence, increasing the expression of PHGDH by the KD might additionally polarize microglia toward anti-inflammatory M2 phenotype, thereby resulting in neuroprotection. Interestingly, the gut microbiome plays a crucial role in serine synthesis (63), thereby increasing serine levels in the brain (60, 64). Thus, the KD likely activates PHGDH, which can be linked to the induction of several neuroprotective and antiseizure effects.

## Conclusion

The treatment of a complex disease such as epilepsy warrants novel treatment approaches, even in an era of ample available ASM (65). Almost 20 years ago, treatments were developed targeting one specific receptor or mechanism (66). In the last decade, however, focus has been directed toward the development of epilepsy treatments based on multiple mechanisms instead of one (4).

The KD is such a therapy modulating various distinct pathways as underlined by a plethora of pre-clinical data (15, 17–20, 24, 56, 67–70). Clinical data are rather scarce; for example, concentrations of ketone bodies in the blood (71), GABA levels in the CSF (20), and *Bacteroidetes* and proteobacteria in the gut (51, 53, 54) have been related to the KD therapy. Hence, future studies should investigate if and how certain pathways can be clinically proven to be impacted by the KD.

Even though different mechanisms of the KD have been reviewed the last few years (15, 17, 18, 20, 56) (i.e., focusing on the primary antiseizure mechanisms), we have compiled a comprehensive overview of the ancillary pathways that are affected by the KD and discussed their pre-clinical and clinical evidence in epilepsy treatment. These include KD-induced changes of the endocrine system, epigenetic control, the gut microbiome, and the serine synthesis *via* PHGDH. Overall, this review and future studies will contribute to the identification of specific pathways of the KD.

## Author Contributions

JS: conceptualization, methodology, validation, formal analysis, investigation, resources, data curation, writing (original draft and review and editing), and visualization. KT: conceptualization, writing (review and editing), and supervision. LL: conceptualization, investigation, writing (review and editing), and supervision. All authors contributed to the article and approved the submitted version.

## Conflict of Interest

LL received grants and is a consultant and/or speaker for Zogenix; LivaNova, UCB, Shire, Eisai, Novartis, Takeda/Ovid, NEL, and Epihunter. KT acknowledges receipt of a mandate of IOF, KU Leuven (IOFm/05/022). The remaining author declares that the research was conducted in the absence of any commercial or financial relationships that could be construed as a potential conflict of interest.

## Publisher's Note

All claims expressed in this article are solely those of the authors and do not necessarily represent those of their affiliated organizations, or those of the publisher, the editors and the reviewers. Any product that may be evaluated in this article, or claim that may be made by its manufacturer, is not guaranteed or endorsed by the publisher.
