# Supplementary material for: The Ketogenic Diet Revisited: Beyond Ketones
Source: Front Neurol. 2021 Jul 30;12:720073. doi: 10.3389/fneur.2021.720073 (PMC8363000; doi:10.3389/fneur.2021.720073)
Supplement: Supplementary file 1 [file Table_1.DOCX]

***SUPPLEMENTARY INFORMATION***

***Search strategy***

We reviewed the current literature using MEDLINE (using PubMed) up to April 2021. The following free and medical subject heading (MeSH) terms were used: ((mechanism) OR (mechanism of action[MeSH Terms])) AND (((ketogenic diet) OR ((ketogenic diet[MeSH Terms]))) OR ((ketogenic diets[MeSH Terms]))). The most recent studies (of the last five years) were selected and abstracts were screened. Eligible studies that focused on the exploration of the mechanism of action of the ketogenic diet were selected. In addition, reference lists were checked of the retrieved studies for reports of other relevant studies.

Our search resulted in 642 studies, of which 294 have been published in the last five years. After screening of the abstracts, 54 studies were used to generate this review. Another 18 general references regarding epilepsy and epilepsy treatment were added, leading to a total of 72 references.

***Supplementary figure***


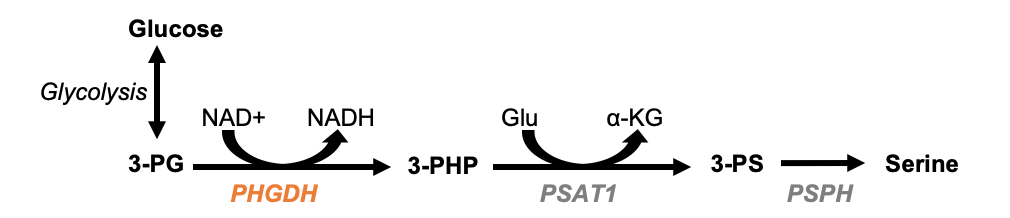


**Figure S1: graphical representation of the *Phosphoglycerate dehydrogenase* pathway.**

Serine can be synthesized via the glycolytic intermediate 3‑phosphoglycerate (3-PG). Phosphoglycerate dehydrogenase (*PHGDH*) is responsible for the oxidation of 3-PG to 3‑phosphohydroxypyruvate (3-PHP). Subsequently, phosphoserine aminotransferase 1 (*PSAT1*) converts 3-PHP into 3‑phosphoserine (3-PS) via the conversion of glutamate (Glu) to α‑ketoglutarate (α‑KG). Next, phosphoserine phosphatase (*PSPH*) catalyzes the hydrolysis of 3-PS to serine.
